# Supplementary material for: Evaluation of critical data processing steps for reliable prediction of gene co-expression from large collections of RNA-seq data
Source: PLoS One. 2022 Jan 28;17(1):e0263344. doi: 10.1371/journal.pone.0263344 (PMC8797241; doi:10.1371/journal.pone.0263344)
Supplement: S1 Fig — Scatterplots are shown for the eight quality measures (see main manuscript and Methods section) based on the top 100 highly correlated genes (X axes) and the top 50 highly correlated genes (A) or the top 200 highly correlated genes (B) (Y axes). Scatterplots show data for 200 randomly selected co-expression networks (out of a total of 7,200 networks). Pearson correlations coefficients (and p-values) are indicated. For each quality measure a high correlation (PCCs between 0.93 and 0.98) was observed, suggesting that the quality measures are robust with regard to the number of highly correlated genes they are based on. (DOCX) [file pone.0263344.s001.docx]

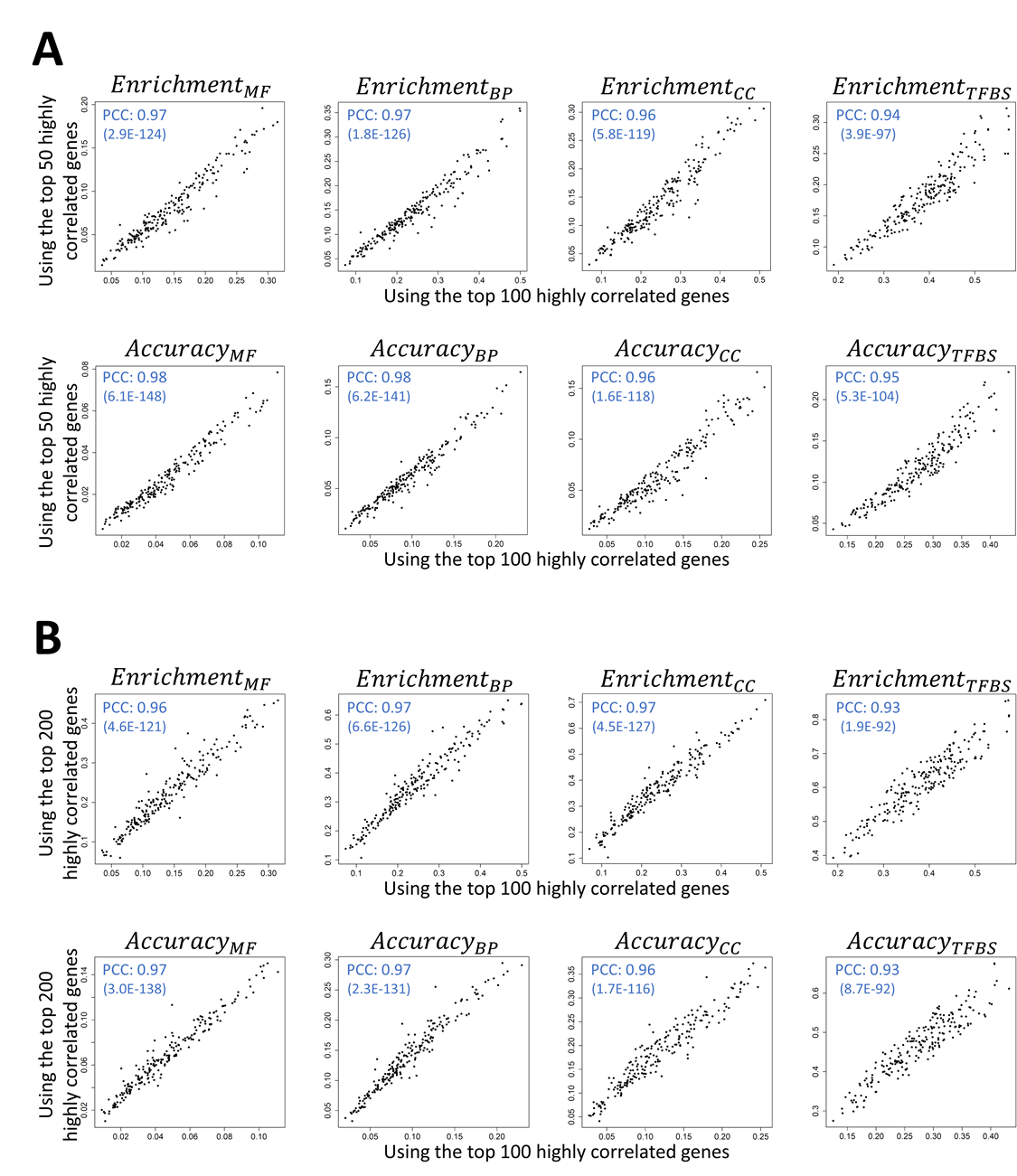


**Supplementary Figure S1: Consistency of the eight quality measures with regard to the number of top correlated genes they are based on.** Scatterplots are shown for the eight quality measures (see main manuscript and Methods section) based on the top 100 highly correlated genes (X axes) and the top 50 highly correlated genes **(A)** or the top 200 highly correlated genes **(B)** (Y axes). Scatterplots show data for 200 randomly selected co-expression networks (out of a total of 7,200 networks). Pearson correlations coefficients (and p-values) are indicated. For each quality measure a high correlation (PCCs between 0.93 and 0.98) was observed, suggesting that the quality measures are robust with regard to the number of highly correlated genes they are based on.
