# Supplementary material for: Evaluation of critical data processing steps for reliable prediction of gene co-expression from large collections of RNA-seq data
Source: PLoS One. 2022 Jan 28;17(1):e0263344. doi: 10.1371/journal.pone.0263344 (PMC8797241; doi:10.1371/journal.pone.0263344)
Supplement: S2 Fig — (A) Proportion of the variance in the eight quality measures explained by the principal components (PCs). The first and second PCs explain 81.4% and 12.1% of the total variance, respectively. (B) Bar plot of the loadings of the first and second PC. (C-D) Scatterplots of PC1 (C) and PC2 (D) (in the X-axes) versus each of the eight individual quality measures (Y-axes). Each plot shows 7,200 dots, each representing a genome-wide gene-gene co-expression network for a cell type or tissue. The Pearson correlation coefficient (PCC) and its p-value are indicated in each plot. (DOCX) [file pone.0263344.s002.docx]

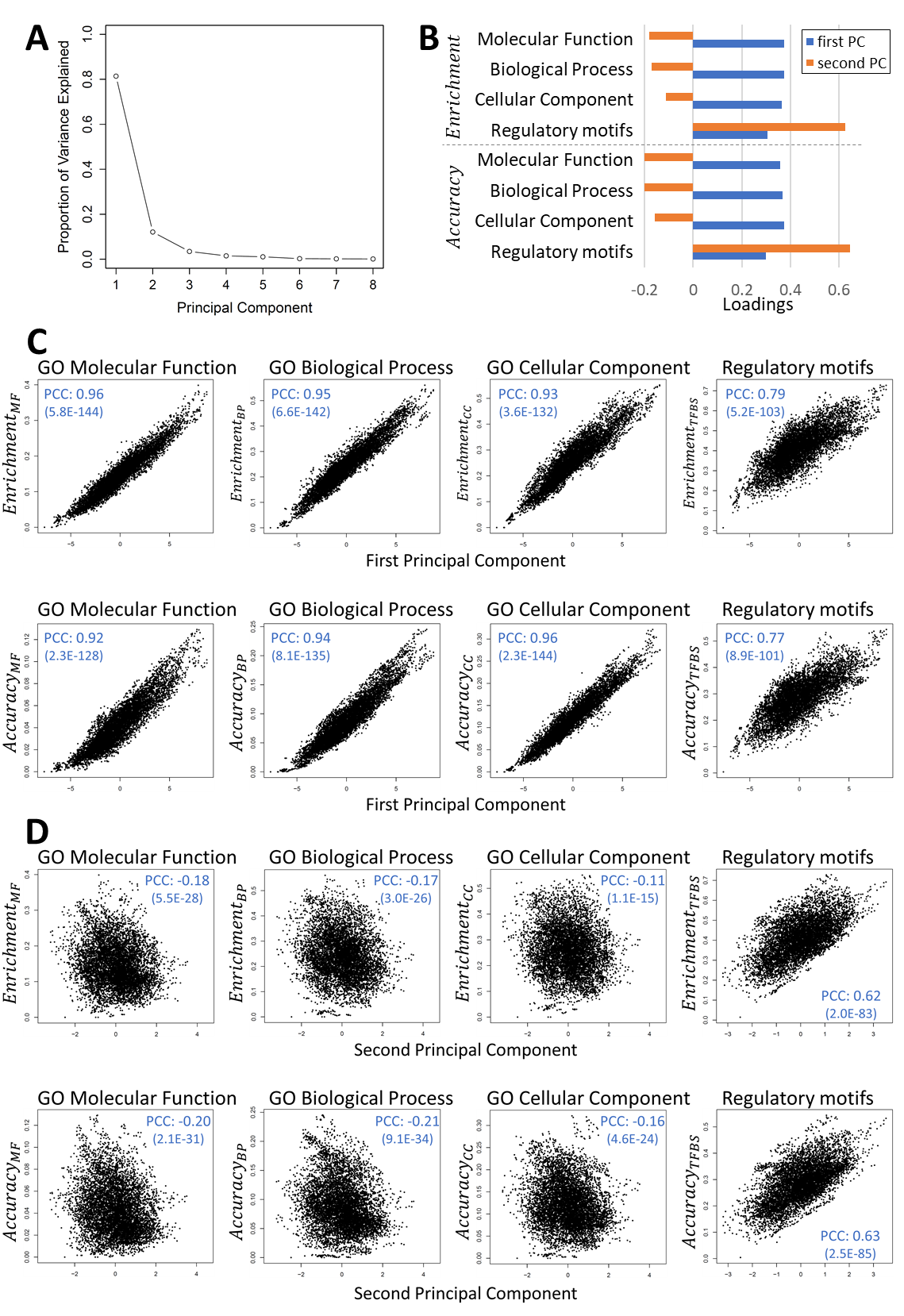


**(previous page) Supplementary Figure S2: Principal Component Analysis of the eight quality measures.** **(A)** Proportion of the variance in the eight quality measures explained by the principal components (PCs). The first and second PCs explain 81.4% and 12.1% of the total variance, respectively. **(B)** Bar plot of the loadings of the first and second PC. **(C-D)** Scatterplots of PC1 **(C)** and PC2 **(D)** (in the X-axes) versus each of the eight individual quality measures (Y-axes). Each plot shows 7,200 dots, each representing a genome-wide gene-gene co-expression network for a cell type or tissue. The Pearson correlation coefficient (PCC) and its p-value are indicated in each plot.
