# Supplementary material for: Evaluation of critical data processing steps for reliable prediction of gene co-expression from large collections of RNA-seq data
Source: PLoS One. 2022 Jan 28;17(1):e0263344. doi: 10.1371/journal.pone.0263344 (PMC8797241; doi:10.1371/journal.pone.0263344)
Supplement: S3 Fig — (A) The distribution of the 7,200 general quality scores, Quality. (B-C) The 8 quality measures of the worst (red), the median (green), and the best (blue) network for GO Molecular Function, Biological Process, Cellular Component and Regulatory motifs in promoter sequences. (B) shows the frequency of enrichment and (C) the accuracy. (DOCX) [file pone.0263344.s003.docx]

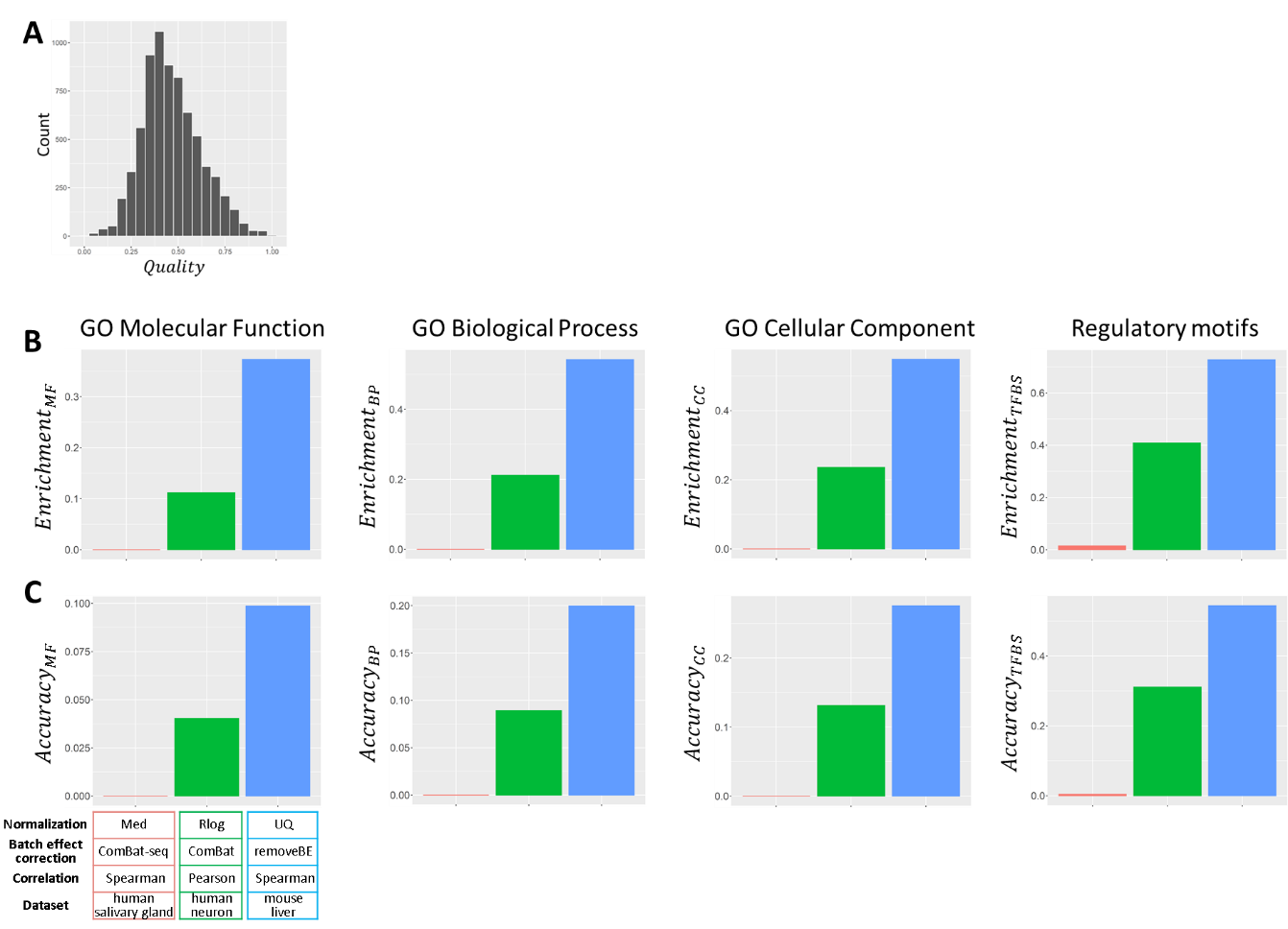


**Supplementary Figure S3: Distribution of the** $\boldsymbol{Quality}$ **score. (A)** The distribution of the 7,200 general quality scores, $Quality$. **(B-C)** The 8 quality measures of the worst (red), the median (green), and the best (blue) network for GO Molecular Function, Biological Process, Cellular Component and Regulatory motifs in promoter sequences. **(B)** shows the frequency of enrichment and **(C)** the accuracy.
