# Supplementary material for: Evaluation of critical data processing steps for reliable prediction of gene co-expression from large collections of RNA-seq data
Source: PLoS One. 2022 Jan 28;17(1):e0263344. doi: 10.1371/journal.pone.0263344 (PMC8797241; doi:10.1371/journal.pone.0263344)

**(next page) Supplementary Figure S4: Overview of the performance of each workflow on each of the 144 datasets.** For each of the 50 workflows the relative performance on each of the 144 datasets is visualized. Workflows are ordered in order of average overall performance as in Fig 2 in the main text. Datasets are ordered according to sample counts. Colors indicate the relative performances of the 50 workflows on each dataset.


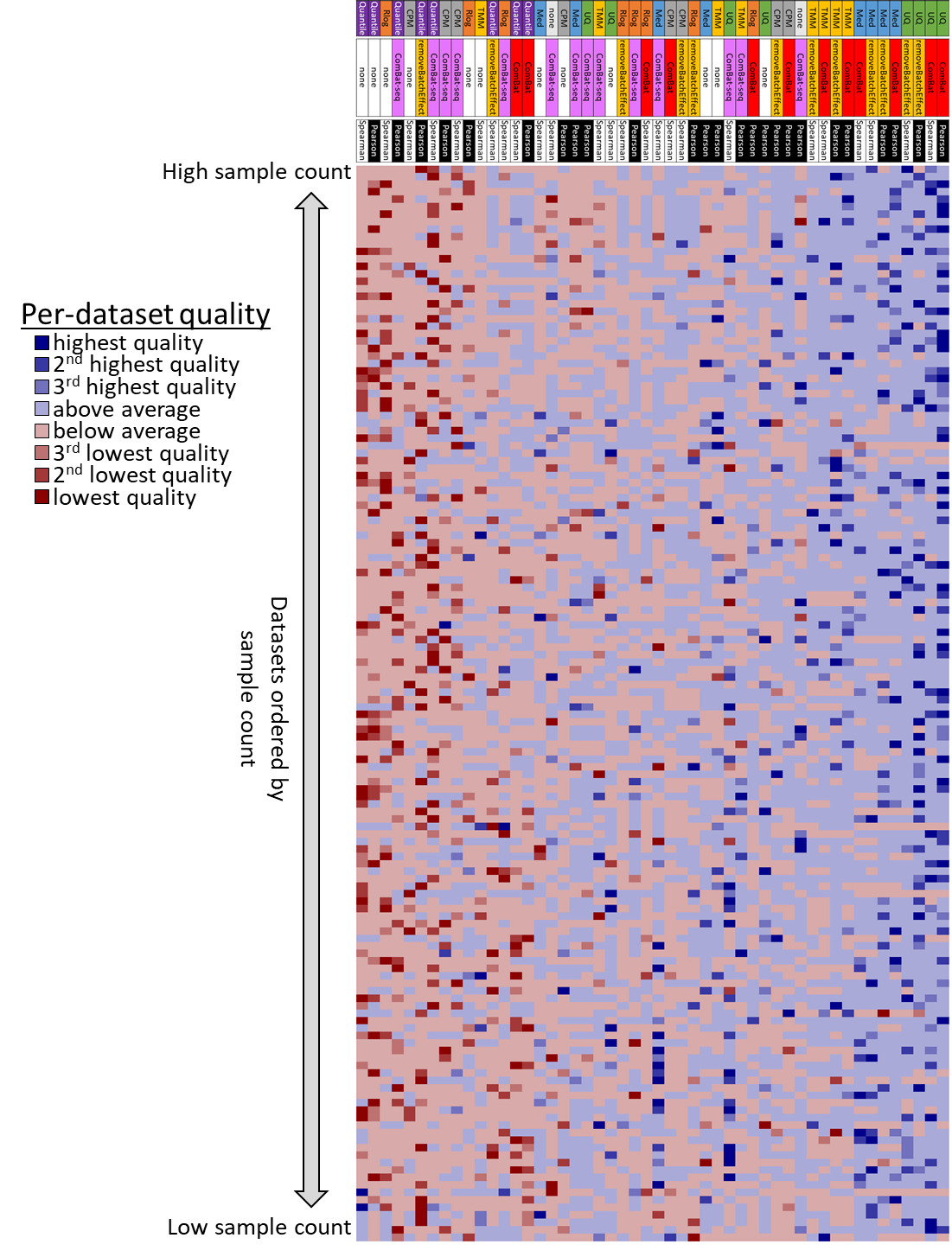

Supplement: S4 Fig — For each of the 50 workflows the relative performance on each of the 144 datasets is visualized. Workflows are ordered in order of average overall performance as in Fig 2 in the main text. Datasets are ordered according to sample counts. Colors indicate the relative performances of the 50 workflows on each dataset. (DOCX) [file pone.0263344.s004.docx]
