# Supplementary material for: Evaluation of critical data processing steps for reliable prediction of gene co-expression from large collections of RNA-seq data
Source: PLoS One. 2022 Jan 28;17(1):e0263344. doi: 10.1371/journal.pone.0263344 (PMC8797241; doi:10.1371/journal.pone.0263344)
Supplement: S5 Fig — The Pearson correlation between the sample count and batch count (both in log10 values) is 0.84. (DOCX) [file pone.0263344.s005.docx]

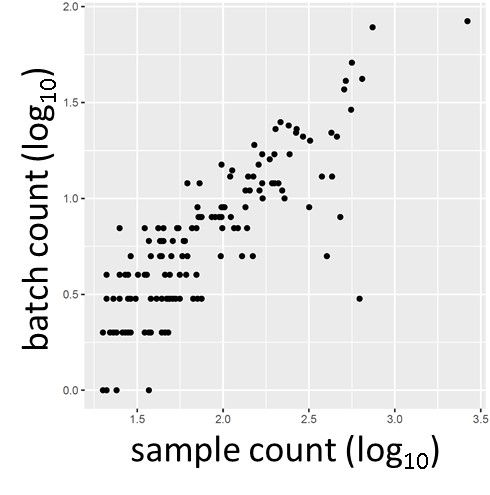


**Supplementary Figure S5: Scatterplot of the sample count and batch count for the 144 cell types and tissues.** The Pearson correlation between the sample count and batch count (both in log_10_ values) is 0.84.
