# Supplementary material for: Evaluation of critical data processing steps for reliable prediction of gene co-expression from large collections of RNA-seq data
Source: PLoS One. 2022 Jan 28;17(1):e0263344. doi: 10.1371/journal.pone.0263344 (PMC8797241; doi:10.1371/journal.pone.0263344)
Supplement: S1 Table — The cell type or tissue, the number of RNA-seq samples, the number of genes included in the final co-expression network, and the number of GO terms tested for the estimation of the network quality is shown. The last column indicates which datasets were included in the validation set. (DOCX) [file pone.0263344.s006.docx]

|  | **tissue or cell type** | **no. of samples** | **no. of genes in co-expression network** | **no. of tested GO terms** | **included in validation set** |
| --- | --- | --- | --- | --- | --- |
| 1 | fibroblast | 645 | 21,070 | 20,531 | Yes |
| 2 | ileum | 623 | 18,655 | 20,778 |  |
| 3 | lymphoblastoid | 481 | 16,687 | 19,899 |  |
| 4 | breast cancer | 427 | 27,783 | 21,042 |  |
| 5 | acute myeloid leukemia | 402 | 24,454 | 20,749 |  |
| 6 | PBMC | 376 | 20,579 | 20,132 |  |
| 7 | lung | 321 | 22,531 | 20,875 | Yes |
| 8 | liver | 292 | 22,937 | 20,926 |  |
| 9 | epithelial cell | 268 | 21,796 | 20,758 |  |
| 10 | brain | 244 | 27,688 | 21,059 |  |
| 11 | pancreas | 228 | 21,400 | 20,834 | Yes |
| 12 | colon | 210 | 20,354 | 20,890 | Yes |
| 13 | B cell | 199 | 21,648 | 20,308 | Yes |
| 14 | monocyte | 193 | 19,557 | 20,029 |  |
| 15 | induced pluripotent stem cell | 187 | 20,283 | 20,728 |  |
| 16 | skeletal muscle | 170 | 18,775 | 20,476 |  |
| 17 | embryonic stem cell | 169 | 22,967 | 20,920 |  |
| 18 | macrophage | 169 | 22,758 | 20,744 | Yes |
| 19 | prostate | 163 | 25,482 | 20,970 |  |
| 20 | colon cancer | 150 | 21,036 | 20,530 |  |
| 21 | bone marrow | 143 | 24,409 | 20,767 |  |
| 22 | CD4 T cell | 135 | 22,510 | 20,414 |  |
| 23 | lung cancer | 135 | 20,883 | 20,763 |  |
| 24 | prefrontal cortex | 129 | 26,077 | 20,921 |  |
| 25 | heart | 113 | 19,468 | 20,569 |  |
| 26 | breast | 111 | 21,977 | 20,861 |  |
| 27 | neural progenitor cell | 98 | 24,969 | 20,835 | Yes |
| 28 | melanoma | 97 | 22,832 | 20,680 |  |
| 29 | placenta | 86 | 19,210 | 20,443 |  |
| 30 | embryonic kidney | 74 | 21,715 | 20,592 |  |
| 31 | neuron | 72 | 25,675 | 20,964 |  |
| 32 | granulocyte | 70 | 19,814 | 19,684 |  |
| 33 | osteosarcoma | 67 | 16,838 | 20,082 |  |
| 34 | adipose tissue | 66 | 19,435 | 20,678 |  |
| 35 | kidney | 62 | 23,379 | 21,025 | Yes |
| 36 | MCF-7 | 59 | 20,334 | 20,080 |  |
| 37 | endothelial cell | 58 | 21,238 | 20,593 |  |
| 38 | HeLa | 56 | 22,011 | 20,484 |  |
| 39 | CD8 T cell | 51 | 19,860 | 19,999 |  |
| 40 | ovarian cancer | 51 | 24,595 | 20,905 |  |
| 41 | cervical cancer | 50 | 23,836 | 20,691 | Yes |
| 42 | lymphoma | 49 | 28,063 | 20,871 |  |
| 43 | hepatocyte | 48 | 15,570 | 19,795 | Yes |
| 44 | neutrophil | 46 | 19,667 | 20,213 |  |
| 45 | salivary gland | 46 | 29,817 | 20,928 |  |
| 46 | HUVEC | 45 | 21,110 | 20,171 |  |
| 47 | keratinocyte | 45 | 20,350 | 20,327 |  |
| 48 | frontal cortex | 44 | 18,232 | 20,511 |  |
| 49 | mammary gland | 44 | 25,746 | 21,017 |  |
| 50 | thyroid | 43 | 23,415 | 20,854 | Yes |
| 51 | esophagus | 41 | 20,439 | 20,821 | Yes |
| 52 | ovary | 38 | 23,973 | 20,905 |  |
| 53 | osteoblast | 37 | 16,689 | 20,179 | Yes |
| 54 | pluripotent stem cell | 35 | 17,358 | 20,252 |  |
| 55 | prostate cancer | 29 | 23,663 | 20,796 |  |
| 56 | cerebellum | 28 | 20,920 | 20,621 |  |
| 57 | dendritic cell | 28 | 21,994 | 20,528 |  |
| 58 | retina | 28 | 18,675 | 20,619 |  |
| 59 | thyroid cancer | 28 | 25,167 | 20,933 |  |
| 60 | CLL | 27 | 19,035 | 19,880 |  |
| 61 | sarcoma | 27 | 24,168 | 21,024 | Yes |
| 62 | adipocyte | 26 | 14,258 | 19,517 |  |
| 63 | mesenchymal stem cell | 25 | 18,070 | 19,971 | Yes |
| 64 | white adipose tissue | 24 | 16,488 | 20,173 | Yes |
| 65 | natural killer cell | 23 | 19,389 | 19,850 |  |
| 66 | cancer | 21 | 26,083 | 20,971 |  |
| 67 | cerebral cortex | 21 | 20,773 | 20,698 |  |
| 68 | neocortex | 20 | 16,623 | 20,405 | Yes |

**Supplementary Table S1: Human datasets.** The cell type or tissue, the number of RNA-seq samples, the number of genes included in the final co-expression network, and the number of GO terms tested for the estimation of the network quality is shown. The last column indicates which datasets were included in the validation set.
