# Supplementary material for: Evaluation of critical data processing steps for reliable prediction of gene co-expression from large collections of RNA-seq data
Source: PLoS One. 2022 Jan 28;17(1):e0263344. doi: 10.1371/journal.pone.0263344 (PMC8797241; doi:10.1371/journal.pone.0263344)
Supplement: S2 Table — The cell type or tissue, the number of RNA-seq samples, the number of genes included in the final co-expression network, and the number of GO terms tested for the estimation of the network quality is shown. The last column indicates which datasets were included in the validation set. (DOCX) [file pone.0263344.s007.docx]

|  | **tissue or cell type** | **no. of samples** | **no. of genes in co-expression network** | **no. of tested GO terms** | **included in validation set** |
| --- | --- | --- | --- | --- | --- |
| 1 | liver | 2,644 | 16,287 | 20,080 |  |
| 2 | embryonic stem cell | 742 | 20,955 | 20,576 |  |
| 3 | embryonic fibroblast | 562 | 17,864 | 20,207 |  |
| 4 | macrophage | 556 | 17,977 | 19,809 | Yes |
| 5 | brain | 518 | 20,893 | 20,674 | Yes |
| 6 | heart | 507 | 18,347 | 20,336 |  |
| 7 | hippocampus | 460 | 19,488 | 20,462 | Yes |
| 8 | cerebellum | 431 | 20,472 | 20,538 |  |
| 9 | cortex | 317 | 19,656 | 20,527 |  |
| 10 | spleen | 266 | 19,057 | 20,378 |  |
| 11 | lung | 241 | 19,246 | 20,594 |  |
| 12 | mammary gland | 221 | 19,174 | 20,614 |  |
| 13 | testis | 216 | 23,122 | 20,766 |  |
| 14 | B cell | 202 | 20,650 | 20,095 |  |
| 15 | CD4 T cell | 199 | 17,300 | 19,738 | Yes |
| 16 | neuron | 161 | 21,232 | 20,610 |  |
| 17 | kidney | 152 | 19,643 | 20,612 |  |
| 18 | whole embryo | 149 | 15,399 | 20,075 | Yes |
| 19 | retina | 140 | 18,522 | 20,381 |  |
| 20 | cardiomyocyte | 138 | 17,309 | 20,303 |  |
| 21 | Th17 | 122 | 15,888 | 19,307 |  |
| 22 | white adipose tissue | 116 | 19,900 | 20,653 |  |
| 23 | CD8 T cell | 110 | 16,718 | 19,510 | Yes |
| 24 | thymus | 102 | 21,358 | 20,800 | Yes |
| 25 | muscle | 100 | 16,188 | 20,041 |  |
| 26 | spinal cord | 99 | 19,828 | 20,575 |  |
| 27 | hematopoietic stem cell | 98 | 21,466 | 20,486 |  |
| 28 | microglia | 98 | 19,901 | 20,309 |  |
| 29 | fibroblast | 95 | 19,727 | 20,349 | Yes |
| 30 | dendritic cell | 90 | 17,107 | 19,651 |  |
| 31 | neocortext | 75 | 14,482 | 19,866 |  |
| 32 | neural stem cell | 75 | 17,587 | 20,092 |  |
| 33 | iPS | 73 | 18,721 | 20,379 | Yes |
| 34 | prefrontal cortex | 71 | 16,031 | 20,154 | Yes |
| 35 | Treg | 71 | 19,567 | 20,076 |  |
| 36 | colon | 70 | 18,570 | 20,540 |  |
| 37 | cerebral cortex | 62 | 17,569 | 20,232 |  |
| 38 | dorsal root ganglion | 61 | 17,996 | 20,507 |  |
| 39 | pro-B | 60 | 15,496 | 19,652 | Yes |
| 40 | myoblast | 59 | 17,415 | 20,068 |  |
| 41 | pre-B | 56 | 16,083 | 19,202 |  |
| 42 | spermatocyte | 55 | 19,841 | 20,341 |  |
| 43 | brown adipose tissue | 54 | 16,610 | 20,060 |  |
| 44 | osteoblast | 53 | 15,999 | 19,937 | Yes |
| 45 | frontal cortex | 49 | 18,596 | 20,501 |  |
| 46 | motoneuron | 48 | 16,020 | 19,991 |  |
| 47 | trophoblast stem cell | 47 | 15,389 | 19,679 |  |
| 48 | AML | 44 | 16,143 | 19,501 |  |
| 49 | astrocyte | 44 | 20,342 | 20,395 |  |
| 50 | spermatid | 43 | 19,840 | 20,113 |  |
| 51 | endothelial cell | 42 | 20,169 | 20,608 |  |
| 52 | midbrain | 38 | 19,159 | 20,511 |  |
| 53 | thymocyte | 38 | 14,956 | 19,465 |  |
| 54 | neural progenitor cell | 37 | 18,891 | 20,396 | Yes |
| 55 | Th0 | 37 | 14,564 | 19,307 |  |
| 56 | small intestine | 36 | 17,929 | 20,411 | Yes |
| 57 | pancreas | 35 | 16,682 | 20,425 |  |
| 58 | skeletal muscle | 35 | 19,382 | 20,342 |  |
| 59 | hepatocyte | 32 | 14,696 | 19,837 | Yes |
| 60 | 3T3 | 31 | 15,091 | 19,470 | Yes |
| 61 | forebrain | 29 | 20,229 | 20,571 |  |
| 62 | placenta | 29 | 16,515 | 20,257 |  |
| 63 | large intestine | 28 | 19,992 | 20,589 |  |
| 64 | duodenum | 27 | 30,937 | 20,945 |  |
| 65 | ileum | 27 | 18,358 | 20,533 |  |
| 66 | oocyte | 27 | 14,937 | 19,793 | Yes |
| 67 | uterus | 26 | 15,813 | 20,303 |  |
| 68 | intestine | 25 | 19,746 | 20,669 | Yes |
| 69 | Th1 | 25 | 17,516 | 19,768 |  |
| 70 | thyroid | 24 | 12,941 | 19,583 | Yes |
| 71 | satellite cell | 23 | 17,247 | 20,059 |  |
| 72 | stomach | 23 | 19,209 | 20,644 |  |
| 73 | mesoderm | 22 | 16,219 | 19,967 |  |
| 74 | olfactory bulb | 21 | 17,698 | 20,199 |  |
| 75 | trophoblast | 21 | 13,038 | 19,407 |  |
| 76 | medullary thymic epithelial cell | 20 | 22,097 | 20,997 |  |

**Supplementary Table S2: Mouse datasets.** The cell type or tissue, the number of RNA-seq samples, the number of genes included in the final co-expression network, and the number of GO terms tested for the estimation of the network quality is shown. The last column indicates which datasets were included in the validation set.
