# Supplementary material for: Evaluation of critical data processing steps for reliable prediction of gene co-expression from large collections of RNA-seq data
Source: PLoS One. 2022 Jan 28;17(1):e0263344. doi: 10.1371/journal.pone.0263344 (PMC8797241; doi:10.1371/journal.pone.0263344)
Supplement: S3 Table — Human and mouse cell types and tissues were randomly divided into 4 folds. Each fold was left out and a linear regression model was trained on the remaining 3 folds. Model 1 is equivalent to the model shown in Table 2 in the main manuscript. For each model, the estimated coefficient (and corresponding p-value) for each parameter is shown. The coefficients estimated in each model are in general consistent with each other. (DOCX) [file pone.0263344.s008.docx]

|  | **Feature** | **Model 1** | **Model 2** | **Model 3** | **Model 4** |
| --- | --- | --- | --- | --- | --- |
|  | **(Intercept)** | -0.150  (5.1E-43) | -0.0563  (2.6E-6) | -0.1169  (9.2E-24) | -0.0855  (1.1E-13) |
|  | **log10(sample count)** | 0.2894  (8.5E-295) | 0.2483  (1.1E-175) | 0.2756  (5.5E-223) | 0.2704  (3.8E-234) |
|  | **log10(batch count)** | -0.0302  (0.00019) | -0.0251  (0.0080) | -0.0200  (0.021) | -0.0231  (0.0070) |
| **species** | **human** | *Baseline* | | | |
|  | **mouse** | 0.0462  (3.0E-39) | 0.0198  (1.5E-7) | 0.0215  (3.7E-10) | 0.0150  (6.5E-5) |
| **normalization** | **Quantile** | *Baseline* | | | |
|  | **Rlog** | 0.0231  (0.00012) | 0.0223  (0.00058) | 0.0210  (0.00032) | 0.0235  (0.00026) |
|  | **CPM** | 0.0318  (1.2E-7) | 0.0358  (3.5E-8) | 0.0302  (2.6E-7) | 0.0362  (2.1E-8) |
|  | **TMM** | 0.0540  (3.0E-19) | 0.0546  (4.7E-17) | 0.0498  (2.1E-17) | 0.0558  (6.6E-18) |
|  | **Med** | 0.0638  (3.9E-26) | 0.0677  (3.0E-25) | 0.0587  (2.0E-23) | 0.0696  (7.6E-27) |
|  | **UQ** | 0.0782  (3.4E-38) | 0.0822  (3.7E-26) | 0.0729  (5.0E-35) | 0.0845  (1.5E-38) |
| **batch effect correction** | **no correction** | *Baseline* | | | |
|  | **removeBatchEffect** | 0.0412  (4.1E-22) | 0.0387  (3.7E-17) | 0.0481  (8.9E-31) | 0.0347  (3.0E-14) |
|  | **ComBat** | 0.0468  (5.4E-28) | 0.0490  (2.5E-26) | 0.0523  (5.0E-36) | 0.0434  (2.5E-21) |
| **correlation measure** | **Pearson** | *Baseline* | | | |
|  | **Spearman** | -0.0107  (0.0019) | -0.0061  (0.10) | -0.0116  (0.00057) | -0.0072  (0.054) |

**Supplementary Table S3: Coefficients and p-values of linear models trained by 4-fold cross-validation.** Human and mouse cell types and tissues were randomly divided into 4 folds. Each fold was left out and a linear regression model was trained on the remaining 3 folds. Model 1 is equivalent to the model shown in Table 2 in the main manuscript. For each model, the estimated coefficient (and corresponding p-value) for each parameter is shown. The coefficients estimated in each model are in general consistent with each other.
