# Supplementary material for: Evaluation of critical data processing steps for reliable prediction of gene co-expression from large collections of RNA-seq data
Source: PLoS One. 2022 Jan 28;17(1):e0263344. doi: 10.1371/journal.pone.0263344 (PMC8797241; doi:10.1371/journal.pone.0263344)
Supplement: S1 Methods — (DOCX) [file pone.0263344.s009.docx]

# Supplementary methods

## Gene expression data

We used the RNASeq-er REST API of the European Bioinformatics Institute (EBI; <https://www.ebi.ac.uk/fg/rnaseq/api/>, [1]) to obtain a list of human and mouse RNA-seq datasets from the European Nucleotide Archive (ENA) with at least 70% of reads mapped to their reference genome, using URLs:

<http://www.ebi.ac.uk/fg/rnaseq/api/tsv/70/getRunsByOrganism/homo_sapiens>

<http://www.ebi.ac.uk/fg/rnaseq/api/tsv/70/getRunsByOrganism/mus_musculus>

Annotation data for each study was downloaded (<http://www.ebi.ac.uk/fg/rnaseq/api/tsv/getSampleAttributesPerRunByStudy/><study ID>), and parsed for “cell type” and “organism part” annotation fields. Annotation terms were manually checked and turned into a list of consistent terms (removing differences and redundancies in spelling and capitalization). This resulted in consistent cell type or tissue annotations for the RNA-seq samples.

For all samples to which we could assign an annotation, raw read counts per gene were downloaded using the URL:

<ftp://ftp.ebi.ac.uk/pub/databases/arrayexpress/data/atlas/rnaseq/studies/ena/><study ID>/<organism>/genes.raw.tsv

where <organism> is either “mus_musculus” or “homo_sapiens”. This read count data has been processed using the iRAP pipeline [2] which includes quality control, alignment to the reference genome using TopHat2 [3], and quantification of the mapped reads per gene using HTSeq [4]. Because we are focusing here on bulk RNA-seq data we excluded single-cell data from the dataset. This resulted in 9,421 samples for human and 12,787 for mouse. After inspection of the distribution of total number of reads per sample, human samples with less than 2.5 million reads and mouse samples with less than 3 million reads were filtered out. Finally, cell type and tissues with less than 20 samples were removed from the further analysis. The final two datasets contained 8,796 human and 12,114 mouse samples, produced by 401 and 630 studies, covering 68 human and 76 mouse cell types and tissues, respectively (S1 and S2 Tables).

## Gene Ontology analysis

Gene-to-GO term association data was obtained from the Mouse Genome Informatics website (<http://www.informatics.jax.org/>) for mouse, and from the EBI database (<ftp://ftp.ebi.ac.uk/pub/databases/GO/goa/HUMAN/goa_human.gaf.gz>) for human. The basic version of the Gene Ontology ([go-basic.obo](http://geneontology.org/docs/download-ontology/#go_basic)) was obtained from GO Consortium website (<http://geneontology.org/>).

GO terms were mapped to their parent terms upward in the GO graph structure. Genes (Entrez IDs) assigned to a particular GO term were also assigned to that term’s parent terms. GO term enrichment in sets of 100 correlated genes (see above) was evaluated using hypergeometric tests: for every GO term the total number of genes associated with the term was compared with the number of genes in the set of 100 genes (Ensembl ids converted to Entrez ids), and a p-value was estimated using a hypergeometric distribution. The Bonferroni correction was used to adjust p-values for multiple testing, and corrected p-values < 0.01 were regarded as significant.

## Transcription factor binding site analysis

Position Weight Matrices (PWMs) were obtained from the JASPAR database (JASPAR_CORE redundant vertebrate PWMs, version of October 2016; 635 PWMs in total) [5]. Promoter sequences (region -500 to +200 around transcription start sites) for all human (hg19/GRCh37) and mouse (mm10/GRCm38) Refseq genes were downloaded using the UCSC Table Browser [6]. We also extracted 10,000 randomly selected regions of the human genome of length 2kb and used them to set a threshold score for each PWM. Threshold scores were set so that each PWM would return on average 1 hit per 5,000 base pairs. A threshold score could be set for 618 PMWs (17 PWMs failed because of low information content).

Vertebrate promoters can be roughly divided into two classes: CpG island-associated promoters and non-CpG island promoters [7,8]. CpG island-associated promoters have on average a higher GC content. To avoid biases caused by differences in GC content and CpG scores, we conducted PWM enrichment analysis as described before [9]. In brief, we classified all human and all mouse promoter sequences into two classes: promoters with high GC content and high CpG scores, and promoters with low GC content and low CpG scores. For each PWM *p*, we calculated *frp,high*, the fraction of high GC content promoters that contain a hit for *p*. Similarly, we calculated *frp,low*, the fraction of low GC content promoters that contain a hit for *p*. For the prediction of enriched PWM motifs in a set of promoters *D*, we counted *hp,D*, the number of sequences that contain a hit for *p*, as well as the number of sequences in *D* that were classified in the high GC content class (*nhigh*) and in the low GC content class (*nlow*), respectively. Finally, using a binomial distribution, we calculated the probability of observing *hp,D* or more hits for *p* in a set of *nhigh* high GC content and *nlow* low GC content sequences, given *frp,high* and *frp,low*. This probability was corrected for multiple testing using the Bonferroni correction, and PWMs with a corrected p-value < 0.01 were considered as significantly enriched in the input set *D*.

# Supplementary references

1. Petryszak R, Fonseca NA, Füllgrabe A, Huerta L, Keays M, Tang YA, et al. The RNASeq-er API-A gateway to systematically updated analysis of public RNA-seq data. Bioinformatics. 2017;33: 2218–2220. doi:10.1093/bioinformatics/btx143

2. Fonseca N, Petryszak R, Marioni J, Brazma A. iRAP - an integrated RNA-seq Analysis Pipeline. bioRxiv. 2014. doi:10.1101/005991

3. Kim D, Pertea G, Trapnell C, Pimentel H, Kelley R, Salzberg SL. TopHat2: accurate alignment of transcriptomes in the presence of insertions, deletions and gene fusions. Genome Biol. 2013;14: R36. doi:10.1186/gb-2013-14-4-r36

4. Anders S, Pyl PT, Huber W. HTSeq-A Python framework to work with high-throughput sequencing data. Bioinformatics. 2015;31: 166–169. doi:10.1093/bioinformatics/btu638

5. Mathelier A, Fornes O, Arenillas DJ, Chen CY, Denay G, Lee J, et al. JASPAR 2016: A major expansion and update of the open-access database of transcription factor binding profiles. Nucleic Acids Res. 2016;44: D110–D115. doi:10.1093/nar/gkv1176

6. Karolchik D, Hinrichs AS, Furey TS, Roskin KM, Sugnet CW, Haussler D, et al. The UCSC Table Browser data retrieval tool. Nucleic Acids Res. 2004;32: D493-6. doi:10.1093/nar/gkh103

7. Lenhard B, Sandelin A, Carninci P. Metazoan promoters: emerging characteristics and insights into transcriptional regulation. Nat Rev Genet. 2012;13: 233–45. doi:10.1038/nrg3163

8. Illingworth RS, Bird AP. CpG islands--’a rough guide’. FEBS Lett. 2009;583: 1713–20. doi:10.1016/j.febslet.2009.04.012

9. Vandenbon A, Dinh VH, Mikami N, Kitagawa Y, Teraguchi S, Ohkura N, et al. Immuno-Navigator, a batch-corrected coexpression database, reveals cell type-specific gene networks in the immune system. Proc Natl Acad Sci U S A. 2016;113: E2393–E2402. doi:10.1073/pnas.1604351113
